# Study on surface enhanced Raman scattering of Au and Au@Al_2_O_3_ spherical dimers based on 3D finite element method

**DOI:** 10.1038/s41598-021-87997-z

**Published:** 2021-04-16

**Authors:** Bao-xin Yan, Yan-ying Zhu, Yong Wei, Huan Pei

**Affiliations:** 1 Key Laboratory for Microstructural Material Physics of Hebei Province, School of Science, Yanshan University, Qinhuangdao, 066004 China; 2 College of Information Science and Engineering, Yanshan University, Qinhuangdao, 066004 China; 3 College of Li Ren, Yanshan University, Qinhuangdao, 066004 China

**Keywords:** Optical physics, Plasma physics

## Abstract

In this paper, the surface enhanced Raman scattering (SERS) characteristics of Au and Au@Al_2_O_3_ nanoparticle dimers were calculated and analyzed by using finite element method (3D-FEM). Firstly, the electric field enhancement factors of Au nanoparticles at the dimer gap were optimized from three aspects: the incident angle of the incident light, the radius of nanoparticle and the distance of the dimer. Then, aluminum oxide is wrapped on the Au dimer. What is different from the previous simulation is that Al_2_O_3_ shell and Au core are regarded as a whole and the total radius of Au@Al_2_O_3_ dimer is controlled to remain unchanged. By comparing the distance of Au nucleus between Au and Au@Al_2_O_3_ dimer, it is found that the electric field enhancement factor of Au@Al_2_O_3_ dimer is much greater than that of Au dimer with the increase of Al_2_O_3_ thickness. The peak of electric field of Au@Al_2_O_3_ dimer moves towards the middle of the resonance peak of the two materials, and it is more concentrated than that of the Au dimer. The maximum electric field enhancement factor 583 is reached at the shell thickness of 1 nm. Our results provide a theoretical reference for the design of SERS substrate and the extension of the research scope.

## Introduction

As a new type of high sensitivity spectral analysis technology, SERS technology is often used to detect objects to be measured at ultra-low concentration or even single molecule level, and can provide the “fingerprint” information of samples very accurately^1^. In recent years, due to its advantages of high sensitivity, high selectivity, little interference by water and fluorescence signal, short detection time and convenient operation, SERS technology has been widely used in material science, medicine, environmental analysis, biomolecule detection and other fields^2–4^.

Since the emergence of SERS technology, various nanostructures made of noble metal nanomaterials have become a research hotspot in this field. Recently, hybrid plasmon nanostructures have attracted more and more attention due to their unique properties and potential nano optical applications^5^. In the experiment, hybrid nanoparticles generally use porous nano sponge as the carrier to infiltrate different materials, thus forming many electromagnetic hot spots in the sponge, which is expected to be used as the substrate for plasmon enhanced spectroscopy and photocatalysis^6,7^. Recently, Yi et al. Introduced ZnO functionalized porous gold nanoparticles. The effective coupling of localized surface plasmon and ZnO excitons shows strong second harmonic emission. Combining them with semiconductor gain materials, it is expected to design efficient coherent nonlinear light sources^8,9^. Agata et al*.* used magnetron sputtering to transfer Ag, Au and Cu nanoparticles onto TiO_2_ and Ti nanotube walls to produce SERS substrate with high activity^10^. He et al^11^. conducted a detailed study on the fluorescence experiments of plasmonic nanostructures in the core–shell of the controlled preparation of the imprinted layer mainly for the detection of plasmon enhanced fluorescence for the specific heteromeric molecules. This paves the way for controllable imprinting of plasmonic nanostructures^12–15^. Kang et al.^16^ reported a method to achieve high-speed and high resolution Raman imaging of living cells by near infrared excitation and high speed verification Raman microscopy using small spherical gold nanoparticles with high narrow nanoscale gap structure. Similarly, the application of SERS technology to molecular detection to improve the sensitivity and high speed and high spatial resolution of live cell Raman images^17–19^ greatly promoted the theoretical exploration of the optimal state of metal particles under different configurations. Therefore, Tian^’^s group of Xiamen University^20^ not only calculated and analyzed the SERS and tip enhanced Raman spectroscopy (TERS) configurations theoretically, but also prepared them by experimental means. It shows the credibility of the theoretical results and broadened the scope of the research field. At present, the methods to calculate the electric field enhancement of nanometer particle configuration mainly include 3D finite difference time domain (3D-FDTD) and 3D-FEM. Anitharaj et al*.*^21^ used the FDTD method to study the local electric field of Ag@SiO_2_ trimer. By gradually breaking the symmetry of the trimer nanospheres, the dependence of two kinds of orthogonal polarization on the plasmon wavelength was analyzed. FDTD discretized algorithm effectively decomposes three-dimensional geometric space into cube subunits. Although FDTD has its own advantages in various applications, FEM is suitable for studying the boundary of curved surface. Due to the mesh algorithm of FEM uses tetrahedrons to smoothly follow the contour of the curve, it provides higher spatial resolution and quantitative accuracy^22^. This feature is particularly important in the study of near-field electromagnetism.

In this paper, 3D-FEM was used to calculate the influence of the wavelength, the angle of incident light, the dimer distance and the radius of the nanoparticle on the electric field enhancement factor. It is found that the distance between the dimers plays a dominant role in electric field enhancement. Al_2_O_3_ is used as the shell layer of the SERS substrate due to its high temperature resistance and oxidation resistance, or the hybridization treatment with precious metals enhances the SERS performance and stability, and the optical properties of Al can tune the electric field peak position^23,24^. After the optimal size model was obtained, Al_2_O_3_ was coated on the surface of Au dimer, and the influence of Al_2_O_3_ thickness on electric field enhancement and Raman enhancement factor were calculated. The results show that the ratio of The Raman enhancement factor of Au@Al_2_O_3_ dimer to that of Au dimer increases with the increase of the thickness of Al_2_O_3_ shell. The calculated results not only reasonably explain the SERS enhanced mechanism of Al_2_O_3_ as the shell material, but also expand the research scope of SERS technology, which have certain guiding significance for the SERS substrate of high sensitivity Al_2_O_3_ shell.

## Theoretical model and calculation method

**Figure 1:**
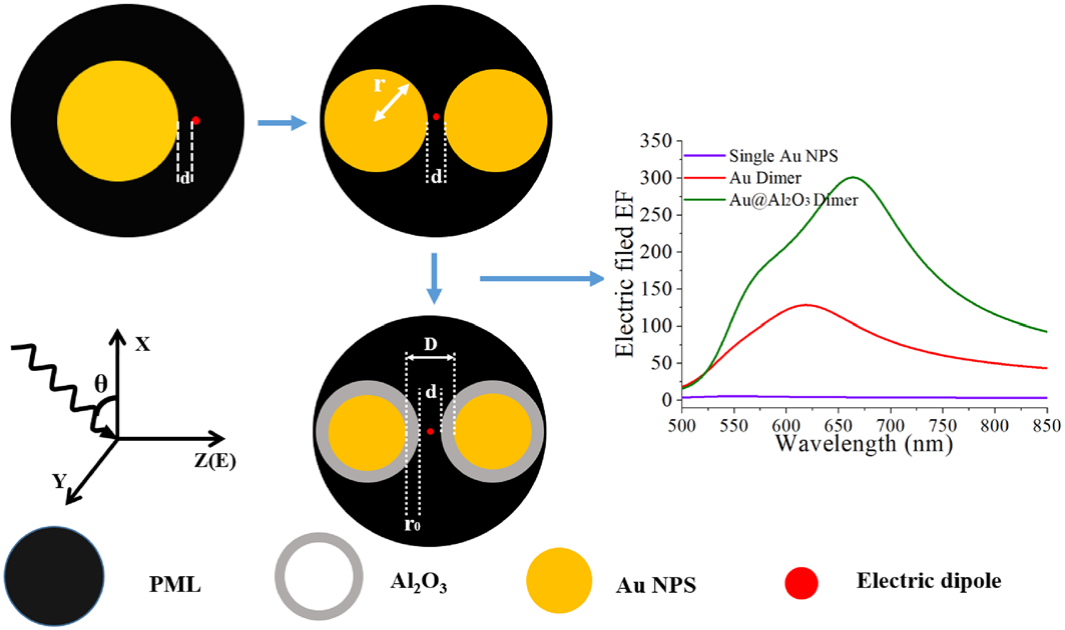
Theoretical model of Au and Au@Al_2_O_3_ dimer. On the right is the comparison diagram of the electric field enhancement factors of Au and Au@Al_2_O_3_ dimer and a single sphere.

Figure 1 demonstrate our theoretical model. COMSOL software is used for simulation calculation, and all materials used in the simulation are from the software library of COMSOL software. The mesh accuracy of the model is selected as an extremely detailed level to ensure the convergence and reliability of the simulation results. Finally, the optical properties of the dimer in a certain wavelength range were studied by wavelength scanning method. The single Au nanosphere, Au dimer and Au@Al_2_O_3_ were calculated by placing the electric dipole in the same position. It is clearly seen from the Fig. 1 that with the further study of configuration, the enhancement factor is constantly increasing. The model of Au@Al_2_O_3_ dimer is composed of two metal spheres, in which the radius of the metal sphere is *r*, the thickness of the shell is *r*_*0*_ and the distance between dimers is *d*. During the modeling process, the wavelength of the plane wave incident to the system is set as *λ*, the angle of the incident light is set as *θ*, and the electric field *E* in the polarization direction is perpendicular to the line between the two centers of the dimer. In the simulation, *E*0(λ) of the incident electric field is set as 1 V/m for ease of calculation. In order to prevent the impact of the reflection field at the boundary on the calculation results, a perfectly matched layer (PML) is used to cover the whole simulation area. Finally, an oscillating point electric dipole is used to represent the molecular radiation in the middle of the metal sphere, and various physical parameters of Au and Au@Al_2_O_3_ dimer are calculated and analyzed theoretically. The localized surface plasmons resonance (LSPR) generated by Au dimer can greatly enhance the Raman signal strength of the target molecule to be measured. Raman enhancement factor is mainly related to two local electric field enhancement factors: the excitation field enhancement factor |*M*(λ*i*)| =|*E*(λ*i*)|/|*E*0(λ*i*)| and the emission field enhancement factor |*M*(λ*s*)| =|*E*(λ*s*)|/|*E*0(λ*s*)| are generated by the interaction of the incident and emitted light fields with the equidistant exciter on the surface of Au. Here, λ*i* and λ*s* represent the wavelength of the incident laser and scattered laser respectively. *E*0(λ) and *E*(λ) represent the intensity of incident and locally enhanced electric fields of the molecules. Finally, the formula for calculating the Raman enhancement factor is^25,26^:

$$RE = {\left| {M(\lambda i)} \right|^2}\left| {M(\lambda s)} \right|{\;^2} = {(\left| {E(\lambda i)} \right|/\left| {E0(\lambda s)} \right|)^2}{(\left| {E(\lambda s)} \right|/\left| {E0(\lambda i)} \right|)^2}$$

where *E*(λ*i*) is the local electric field intensity, and *E*(λ*s*) is the field intensity of the radiation light field with the radiation wavelength λ*s*. The radius of Au@Al_2_O_3_ dimer is calculated as follows: *R* = *r* + *r*_*0*_. Where *R* is the radius of Au@Al_2_O_3_ dimer, *r* is the radius of the Au nucleus, and *r*_*0*_ is the thickness of Al_2_O_3_ layer. The medium in the whole simulated area is air, and *d* represents the distance between the dimers. Then, the SERS performance of Au dimers and Au@Al_2_O_3_ dimers was compared by wrapping Al_2_O_3_ outside Au nucleus. The distance between Au nanospheres of Au@Al_2_O_3_ dimer is defined as: *D* = 2*r*_*0*_ + *d*. Where, *D* is the distance between Au@Al_2_O_3_ dimer Au nanospheres, *r*_0_ is the thickness of Al_2_O_3_ layer, and *d* is the distance between Au@Al_2_O_3_ dimer.

As can be seen from the right side of Fig. 1, compared with a single Au nanosphere, the nanoscale cavity formed between Au dimers makes the electric field more localized, thus forming a highly localized nanoscale spatial resolution. Then Au@Al_2_O_3_ dimer because of Al_2_O_3_ shell indirectly shorten the distance between the dimers, so that the electric field enhancement is improved again.

## Results and discussion

**Figure 2:**
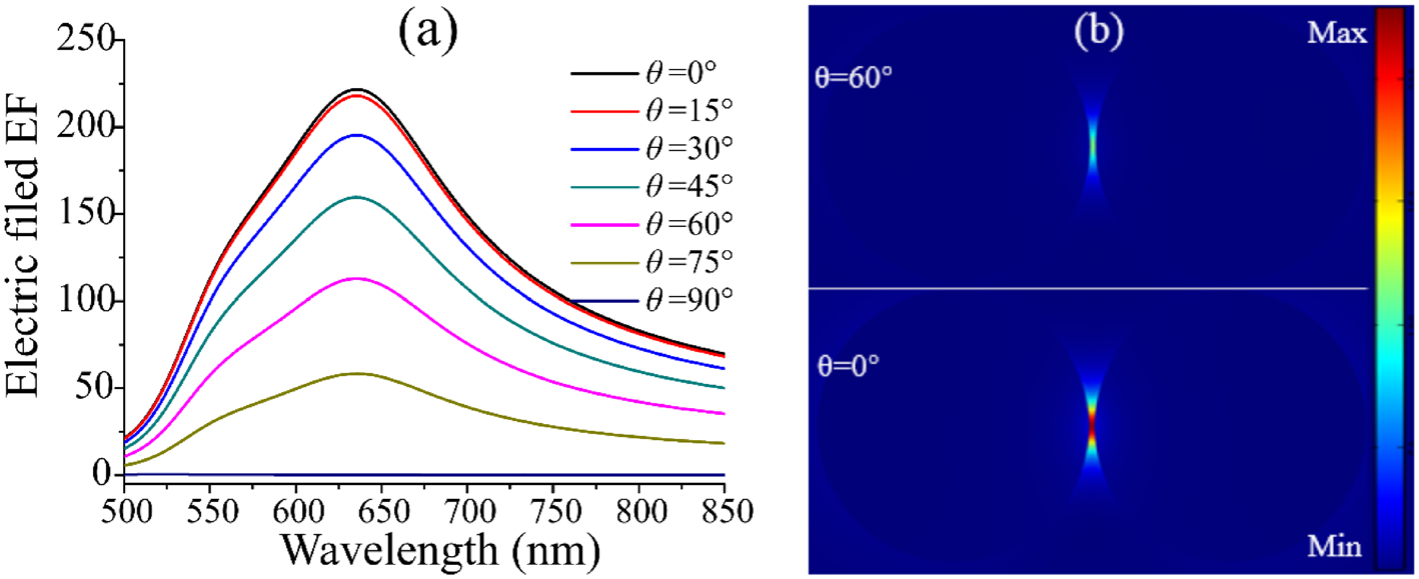
(**a**) The influence of the angle of incident light on the surface enhanced local electric field of Au dimer. Here we set *d* = 2 nm, *r* = 50 nm. (**b**) The electric field distribution at *θ* = 60° and *θ* = 0° with the excitation wavelength of 635 nm. The two electric field distributions are drawn on the same scale.

In order to compare the influence of Al_2_O_3_ shell on the SERS performance of Au dimer, we firstly analyzed the influence of Au spherical dimer on the SERS performance. The radius of Au nanospheres is set as 50 nm, and the distance between the two Au nanospheres is set to 2 nm. Selected light source is linearly polarized plane wave, and the measurement was made every 15° from the X-axis to the Z-axis, so as to discuss its influence on the enhancement performance of the system, as shown in Fig. 2. Figure 2a shows that the electric field enhancement factor decreases gradually when the incident angle increases from 0° ~ 90°. It is noticed that, the wavelength of the peak position is kept 635 nm and does not change with the angle. This because the light absorption property of the material is independent of the change of the incident angle, but related to the configuration of Au dimer. Figure 2b shows the field distribution of Au dimer with the radius of 50 nm and distance of 2 nm under the excited wavelength of 635 nm. The hot spot on the electric field is greatly enhanced and localized area to the nanometer gap, because the electromagnetic field energy in the resonance state is effectively converted into collective vibration energy of free electrons on the metal surface. The red area represents a relative stronger electric field, which demonstrate that the electromagnetic field is enhanced and limited within the scope of the metal surface. Moreover, it can be seen that the electric field strength of dimer gap at *θ* = 0° is significantly higher than that at *θ* = 60° on the same scale, which is because vertical incidence maximizes the vertical field component^27^.

**Figure 3:**
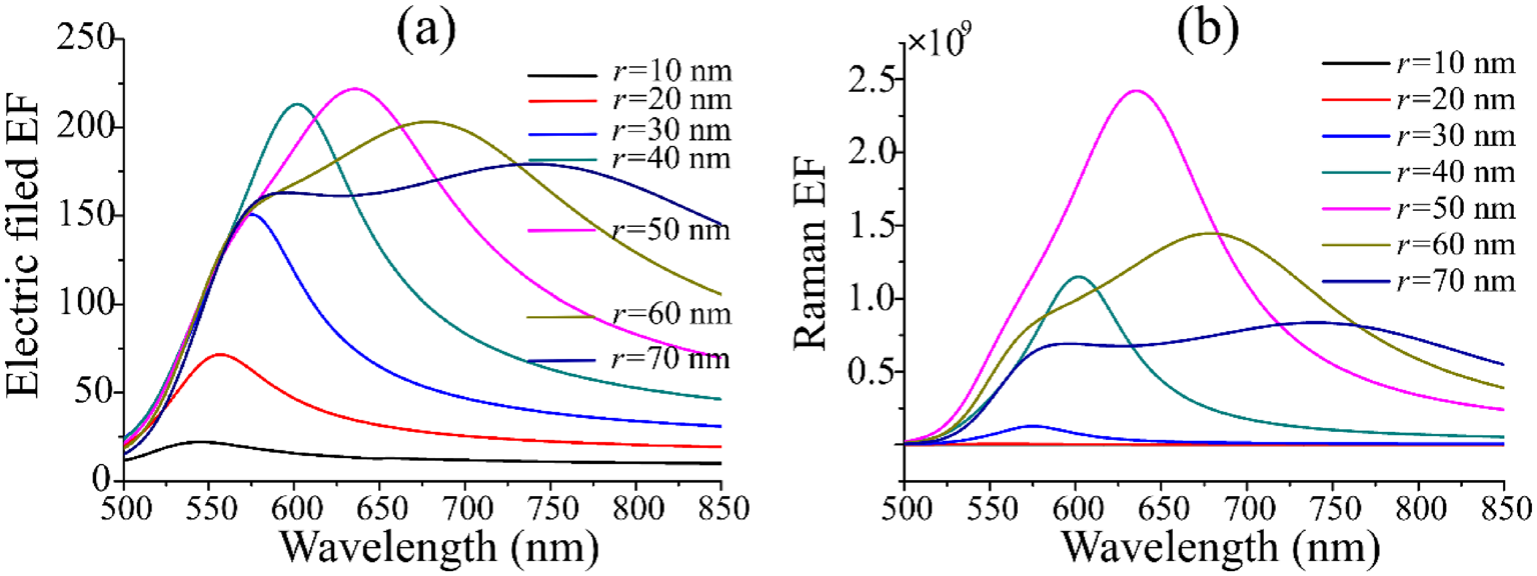
The dependence of the electric field enhancement factor (**a**) and Raman enhancement factor (**b**) of Au dimer on with the variation of incident light wavelength at different radius. Here the incident angle is set as 0°, the distance of Au nanoparticles is 2 nm, and the excitation wavelength is set as 635 nm.

In order to optimize the local electric field and the Raman enhancement, the radius of Au nanospheres should be considered, as shown in Fig. 3. By fixing the angle of incidence *θ* = 0° and the distance between nanoparticles *d* = 2 nm, the radius of Au dimer is calculated in the range from 10 to 70 nm. It can be clearly seen in Fig. 3a that the electric field enhancement factor is very small when *r* = 10 nm, and there are the obvious resonance peaks for the electric field enhancement between 20 and 70 nm. With the increase of the radius, the peak value of the electric field enhancement factor gradually increases and then decreases. When *r* = 50 nm and *λi* = 635 nm, the electric field enhancement reaches the maximum value of around 220, and then the peak begins to decline. In the calculation of Raman enhancement factor, it is found that the electric field enhancement factor of Au dimer with *r* = 70 nm is basically the same in the wavelength range of 575 ~ 750 nm, which makes the selection of excitation wavelength wider and the application range of Au dimer wider. With the increase of radius, the peak value of electric field enhancement factor has obvious red shift. The red shift of the peak is mainly due to the Au nuclear on both sides of the opposite sign of surface charge between the restoring force of abate^28^. For the larger Au nuclear radius, the distance between the surface charge increase results in the decrease of the interaction of the dimer. Therefore, the restoring force between them will be abated, resulting in a red shift phenomenon of the LSPR peak. Figure 3a shows that the electric field enhancement factor is the largest with the excited wavelength of 635 nm, which is used as the excitation wavelength to calculate the Raman enhancement factor with different radius according to formula (1), as shown in Fig. 3b. When *r* = 50 nm, the Raman enhancement factor achieve 10^9^ at around 535 ~ 675 nm, and the maximum Raman enhancement factor is 2.4 × 10^9^ at 635 nm, which will continue to increase with subsequent structural optimization. In the simulation process, it is assumed that the incident wavelength is 635 nm, that is, the Stokes scattering wavelength in Raman scattering should be greater than 635 nm. Therefore, *r* = 60 nm Raman enhancement factor is larger in the range from 635 to 735 nm, and thus the stronger molecular SERS signals can be obtained. However, it is worth noting that when *r* = 40 nm, due to its maximum enhancement factor around 600 nm, it is conducive to the measurement of An-Stokes scattered waves^29^.

**Figure 4:**
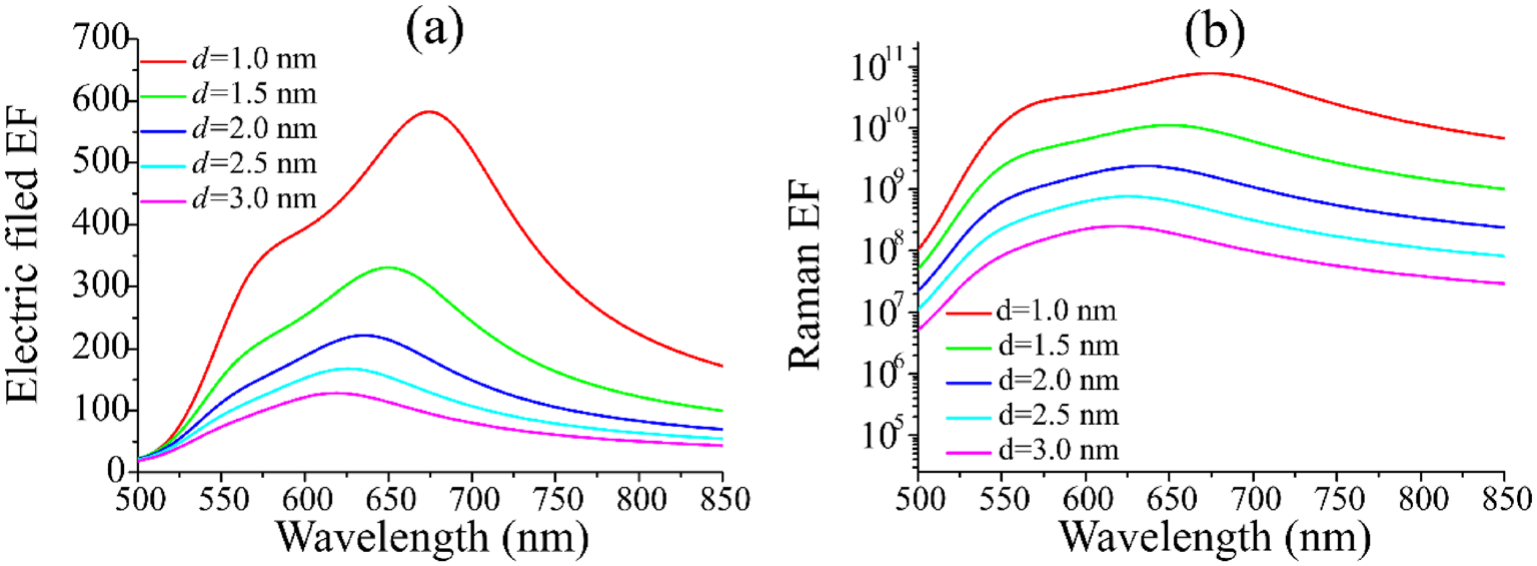
The relation curves of electric field intensity enhancement factor (**a**) and Raman enhancement factor (**b**) with the variation of incident light wavelength at different distance between Au dimers. The incident angle is 0°, the radius of Au nanospheres particle is 50 nm, and the excitation wavelength of Raman enhancement factor is 635 nm.

In order to find better enhancement effect for Au dimers, the effect of the distance between Au dimers on the electric field was calculated with the radius of 50 nm and the incident light angle of 0°, as shown in Fig. 4. With the decrease of the distance between dimers, the peak value of the electric field enhancement factor appears obvious red shift, and it gradually increases with the decrease of the distance from 3 to 1 nm. However, when the distance continues to be reduced to 1 nm, the enhancement factor has a multiple enhancement compared with other distance in the whole simulation range, and reaches the maximum value of around 583 under the excited wavelength of 675 nm. The peak value of enhancement factor is 1.5 ~ 3 times for the other peaks. The intensity of the electric field decreases slightly at 600 ~ 650 nm and then peaks, probably due to the interference of incident light with the scattered radiation^30^. Figure 4b also calculates the Au dimer Raman enhancement factor at excitation wavelength of 635 nm. At *d* = 3 nm, the Raman enhancement factor is generally lower than other distance enhancement factors, but the peak value of the Raman enhancement factor is also up to 10^8^, which can meet general experimental requirements of molecule characterization and recognition. The peak value of Raman enhancement factor is above 10^9^ when the gap distance is the range from 1 to 2 nm, especially for *d* = 1 nm, the maximum value of Raman enhancement factor reaches 7.8 × 10^10^.

**Figure 5:**
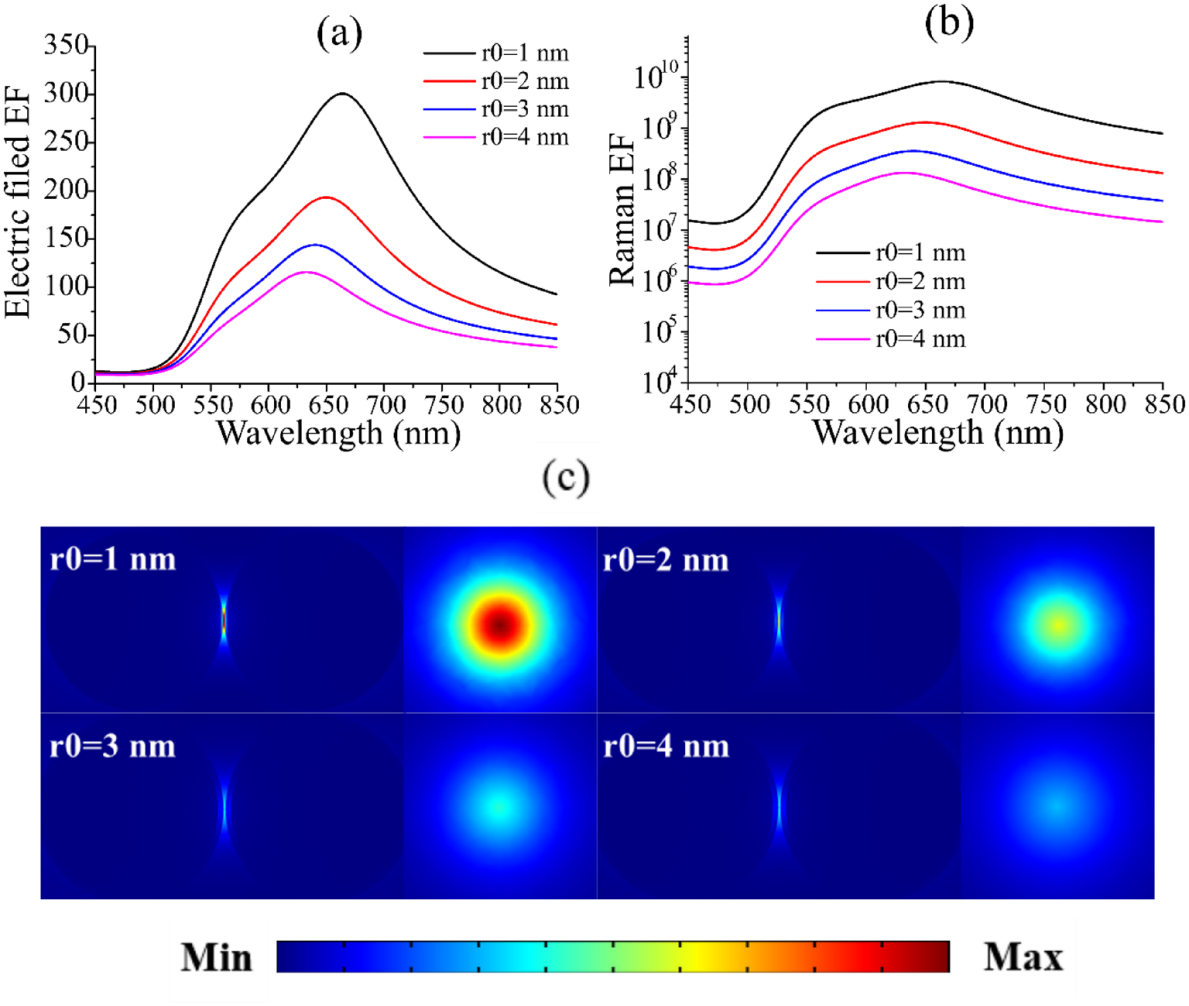
The relation curve of electric field enhancement (**a**) and Raman enhancement factor (**b**) covering different Al_2_O_3_ thickness with the change of incident light wavelength. (**c**) Is the side view and top view of electric field distribution with different thickness Al_2_O_3_ (r_0_ = 1 ~ 4 nm). The excitation wavelength of the Raman enhancement factor and the electric field distribution diagram are both 665 nm. The distance between Au@Al_2_O_3_ dimer is fixed at 1 nm, and the radius R is set to 50 nm.

For the spherical dimer structure, the influence of radius change on the position of the electric field peak is greater than that of the distance. For Au@Al_2_O_3_ core–shell structure, the radius is R = 50 nm. Figure 5a,b shows the curve of electric field enhancement factor and Raman enhancement factor with the change of incident light wavelength in the case of Au@Al_2_O_3_ dimer with distance *d* = 1 nm and total radius *R* = 50 nm. With the increase of the thickness of Al_2_O_3_, the peak value of the electric field enhancement factor gradually decreases and shows a blue shift, which has certain guiding significance to the selection of SERS substrate material and the selection of excitation wavelength. Under the condition of constant radius, Fig. 5c clearly shows from the side diagram and top view of the electric field distribution that the electric field will gradually decrease with the gradual increase of the thickness of Al_2_O_3_. This is mainly because the thickness of the shell increases the distance between Au nuclei^31^. Depending on Su's^32^ paper, the resonance spectrum of Al is in the near ultraviolet spectrum, while that of Au is in the visible spectrum. Therefore, it is speculated that the Al_2_O_3_ covering the surface of the Au nucleus leads to the blue shift of the electric field enhancement factor. Compared with the Au dimer in Fig. 4a, the peak value of the electric field enhancement factor in Au@Al_2_O_3_ dimer is relatively concentrated. This led us to speculate whether the core–shell structure of two different materials would cause the resonance peak to move towards the resonance peak of the other material and eventually reach the tuning of the formants.

**Figure 6:**
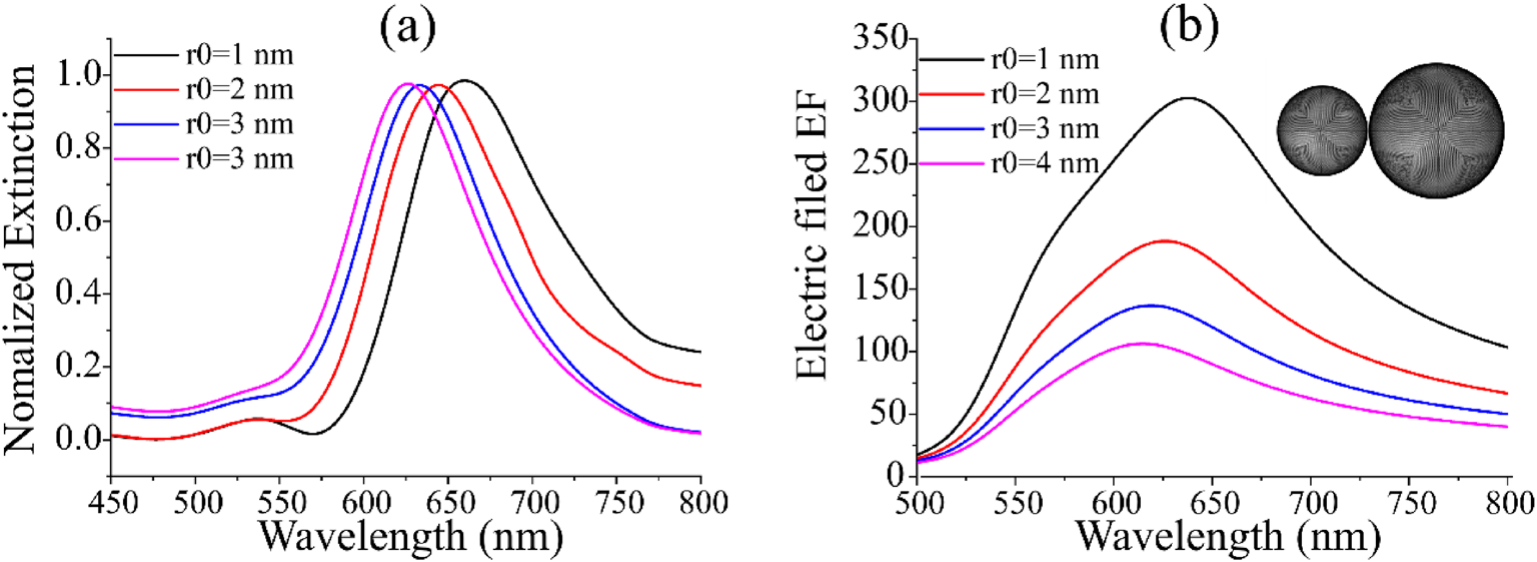
(**a**) Au@Al_2_O_3_ extinction spectrum of dimer varying with thickness of Al_2_O_3_ at the same radius. The data were normalized. Au@Al_2_O_3_ dimer has a radius of 50 nm and a distance of 1 nm. (**b**) Au@Al_2_O_3_ dimer curve of electric field enhancement factor of different shell thickness with wavelength of incident light. The dimer radius are 40 nm and 60 nm, respectively.

In order to study the optical properties of Au@Al_2_O_3_ dimer more clearly, 3D-FEM was used to simulate the extinction spectrum. Figure 6a shows the normalized extinction spectra of Au@Al_2_O_3_ dimer with different shell thickness at the same radius. Au@Al_2_O_3_ dimer has a radius of 50 nm and a distance of 1 nm. The peak value of extinction spectrum in Fig. 6a is basically consistent with the peak value of electric field in Fig. 5a. The extinction spectra show that the peak position shifts blue with the increase of shell thickness. This may be due to the inclusion of Al_2_O_3_. Extinction spectra show that the properties of the structure itself change with the change of shell thickness, but the distance between the surrounding medium and the dimer should be taken into account to calculate the electric field enhancement factor between the dimer. Extinction spectra more clearly show the influence of the change of shell thickness on the position of electric field peak. Figure 6b shows the effect of Au@Al_2_O_3_ dimer with different radius on the electric field enhancement factor. The radius are 40 nm and 60 nm, respectively, as shown in the illustration in Fig. 6b. It can be seen that compared with the Au@Al_2_O_3_ dimer with radius of 50 nm, the electric field enhancement trend is basically the same, except for the blue shift of the electric field peak.

**Figure 7:**
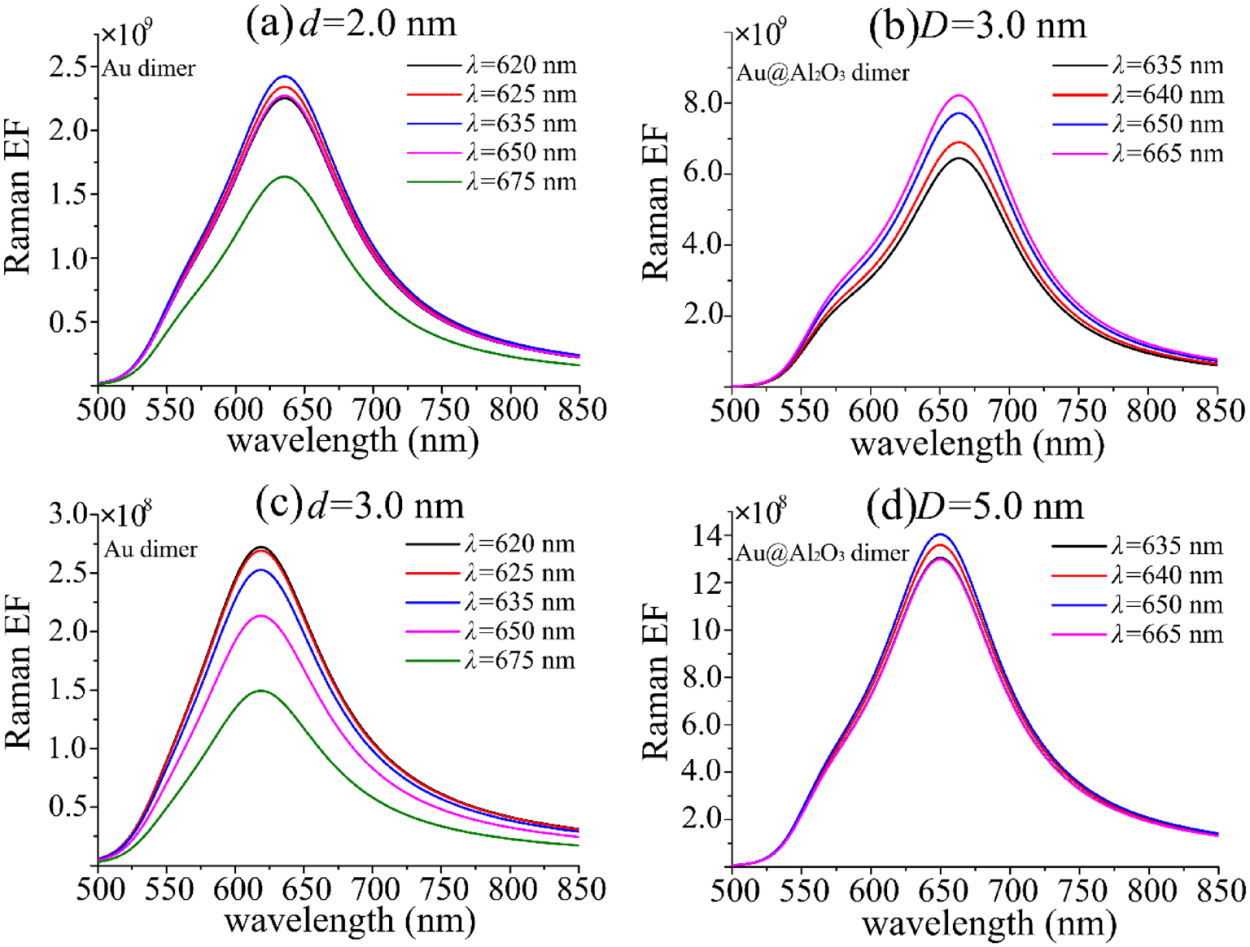
(**a**,**c**) and (**b**,**d**) respectively represent the comparison of Raman enhancement factor curves of Au and Au@Al_2_O_3_ dimers with different excitation wavelengths at different distances, with a radius of 50 nm and incident light angle of 0°. The distance between Au@Al_2_O_3_ dimer is set as *d* = 1 nm, and the thickness of Al_2_O_3_ shell is *r*_0_ = 1 nm and 2 nm respectively, so the distance between Au@Al_2_O_3_ dimer Au core is *D* = 3 nm and 5 nm.

Figure 7 shows the comparison of Raman enhancement factors at different distances of Au and Au@Al_2_O_3_ dimers corresponding to different excitation wavelengths. According to Eq. (1), the Raman enhancement factor is obtained by multiplying the square of the excitation field enhancement factor and the square of the transmitting field enhancement factor. SERS performance of Au and Au@Al_2_O_3_ dimer was compared by changing excitation wavelength. At Au dimers *r* = 50 nm, the spectral position corresponding to the maximum electric field enhancement factor with distance from 1 to 3 nm is selected as the excitation spectrum, i.e. *λex* = 620 nm, 625 nm, 635 nm, 650 nm, 675 nm and the Raman enhancement factor with distance *d* = 2 nm, 3 nm is calculated. Figure 7b,d respectively represent the spectral position corresponding to the maximum electric field enhancement factor of Au@Al_2_O_3_ at *R* = 50 nm, dimer distance *d* = 1 nm, shell thickness *r*_0_ = 1 ~ 4 nm as the excitation spectrum. At *λ*ex = 635 nm, 640 nm, 650 nm, 665 nm, the Raman enhancement factors of Au@Al_2_O_3_ dimer at *D* = 3 nm and 5 nm are calculated. From Fig. 7b,c, it can be seen that when the Au surface distance of the dimer is the same, the Au dimer can indirectly shorten the distance between the dimers by the coating thickness of 1 nm Al_2_O_3_, and the Raman enhancement factor. It is an order of magnitude higher than Au dimer. By comparing Fig. 7c,d, even the Au surface distance of Au@Al_2_O_3_ dimer is 5 nm, the Raman enhancement factor is higher than that of Au dimer with 3 nm distance. Therefore, it is quite meaningful to wrap Al_2_O_3_ material on the Au core shell. At the same distance, there is a certain difference in the Raman enhancement factor under different excitation wavelengths, but it is basically in an order of magnitude, which also shows that the Au dimer has a wider excitation spectrum under different distance, making its application scope wider. Figure 7a,c show that the effect of excitation wavelength on Raman enhancement factor gradually increases with the increase of the distance of Au dimer. But Fig. 7b,d shows that Au@Al_2_O_3_ With the increase of Au surface distance, the effect of excitation wavelength on Raman enhancement factor decreases gradually, and the wide excitation spectrum also makes the application range of the structure itself wider^33^. The electric field peak value of Au@Al_2_O_3_ dimer is more concentrated, which makes it easier to determine the excitation wavelength when Au@Al_2_O_3_ is used as the substrate material, and provides theoretical support for the experiment. It can be found from the Fig. 7 that slight changes in each configuration will change the corresponding optimal excitation wavelength, so the theoretical simulation is very meaningful. The results show that the Raman enhancement of Au@Al_2_O_3_ dimer is very sensitive to distance, and the wrapped Al_2_O_3_ can transfer the Raman enhancement of Au core well. With the progress of subsequent manufacturing methods, ultra-high sensitivity SERS signals were provided for living cell detection and biological imaging.

## Conclusion

In this paper, the effects of electric field enhancement and Raman enhancement of the molecular system in the middle of Au and Au@Al_2_O_3_ dimer are studied by using 3D-FEM. The results show that the enhancement of SERS depends on the angle and wavelength of incident light, the radius of Au nanoparticles and the distance between dimers, but the distance between dimers dominates. When *θ* = 0°, *r* = 50 nm and *d* = 1 nm, the localized surface plasmons resonance coupling in the Au dimer nanometer gap produces a maximum electric field enhancement factor of 583, and reaches the maximum Raman enhancement factor of 1.55 × 10^11^ at the excitation wavelength of 675 nm. The distance between Au dimers is indirectly shortened and the electric field factor is improved by wrapping the inert material of Al_2_O_3_. The introduction of Al_2_O_3_ as the wrapping material in the experiment also avoids the direct contact between Au core and the object to be measured, and ensures that the SERS signal comes from the object to be detected. The results demonstrate that the ratio of the Raman enhancement factor of Au@Al_2_O_3_ dimer to that of Au dimer increases with the increase of the thickness of Al_2_O_3_ shell. This is because the refractive index of Al_2_O_3_ is greater than that of air. Under the condition that Au core is at the same distance, the thickness of Al_2_O_3_ also determines the thickness of the larger refractive index region. Al_2_O_3_ has added to Au nucleus to adjust the maximum spectral peak position of LSPR coupling and make the peak position become centralized. The theoretical results have good guiding significance for studying the internal mechanism of various SERS detection with high sensitivity.
